# Case series of glans injuries during voluntary medical male circumcision for HIV prevention — eastern and southern Africa, 2015–2018

**DOI:** 10.1186/s12894-020-00613-6

**Published:** 2020-04-25

**Authors:** Todd J. Lucas, Carlos Toledo, Stephanie M. Davis, D. Heather Watts, Joseph S. Cavanaugh, Valerian Kiggundu, Anne G. Thomas, Elijah Odoyo-June, Collen Bonnecwe, Tintswalo Hilda Maringa, Enilda Martin, Ambrose Wanyonyi Juma, Sinokuthemba Xaba, Shirish Balachandra, Jotamo Come, Marcos Canda, Rose Nyirenda, Wezi Msungama, James Odek, Gissenge J. I. Lija, Erick Mlanga, James Exnobert Zulu, Heidi O’Bra, Omega Chituwo, Mekondjo Aupokolo, Denis A. Mali, Brigitte Zemburuka, Kananga Dany Malaba, Onkemetse Conrad Ntsuape, Jonas Z. Hines

**Affiliations:** 1 Epidemic Intelligence Service, Centers for Disease Control and Prevention, Atlanta, GA USA; 2 Division of Healthcare Quality Promotion, Centers for Disease Control and Prevention, Atlanta, GA USA; 3 Division of Global HIV and Tuberculosis, Centers for Disease Control and Prevention, Atlanta, GA USA; 4 U.S. Office of the Global HIV/AIDS Coordinator, Washington, D.C USA; 5 Office of HIV/AIDS, U.S. Agency for International Development, Washington, D.C USA; 6 Defense Health Agency, Department of Defense, San Diego, CA USA; 7 Division of Global HIV and Tuberculosis, Centers for Disease Control and Prevention, Nairobi, Kenya; 8 National Department of Health, Pretoria, South Africa; 9 Division of Global HIV and Tuberculosis, Centers for Disease Control and Prevention, Pretoria, South Africa; 10 U.S. Agency for International Development, Pretoria, South Africa; 11 National AIDS and STI Control Program, Ministry of Health, Nairobi, Kenya; 12 Ministry of Health and Child Care, Harare, Zimbabwe; 13 Division of Global HIV and Tuberculosis, Centers for Disease Control and Prevention, Harare, Zimbabwe; 14 Ministry of Health, Maputo, Mozambique; 15 Division of Global HIV and Tuberculosis, Centers for Disease Control and Prevention, Maputo, Mozambique; 16 Ministry of Health, Lilongwe, Malawi; 17 Division of Global HIV and Tuberculosis, Centers for Disease Control and Prevention, Lilongwe, Malawi; 18 U.S. Agency for International Development, Lilongwe, Malawi; 19 National AIDS Control Program, Ministry of Health, Community Development, Gender, Elderly and Children, Dar es Salaam, Tanzania; 20 U.S. Agency for International Development, Dar es Salaam, Tanzania; 21 Ministry of Health, Lusaka, Zambia; 22 U.S. Agency for International Development, Lusaka, Zambia; 23 Division of Global HIV and Tuberculosis, Centers for Disease Control and Prevention, Lusaka, Zambia; 24 Ministry of Health and Social Services, Windhoek, Namibia; 25 U.S. Agency for International Development, Windhoek, Namibia; 26 Division of Global HIV and Tuberculosis, Centers for Disease Control and Prevention, Windhoek, Namibia; 27 Division of Global HIV and Tuberculosis, Centers for Disease Control and Prevention, Gaborone, Botswana; 28 Ministry of Health and Wellness, Gaborone, Botswana

**Keywords:** Male circumcision, Intraoperative complications, HIV, Amputation

## Abstract

**Background:**

Male circumcision confers partial protection against heterosexual HIV acquisition among men. The President’s Emergency Plan for AIDS Relief (PEPFAR) has supported > 18,900,000 voluntary medical male circumcisions (VMMC). Glans injuries (GIs) are rare but devastating adverse events (AEs) that can occur during circumcision. To address this issue, PEPFAR has supported multiple interventions in the areas of surveillance, policy, education, training, supply chain, and AE management.

**Methods:**

Since 2015, PEPFAR has conducted surveillance of GIs including rapid investigation by the in-country PEPFAR team. This information is collected on standardized forms, which were reviewed for this analysis.

**Results:**

Thirty-six GIs were reported from 2015 to 2018; all patients were < 15 years old (~ 0·7 per 100,000 VMMCs in this age group) with a decreasing annual rate (2015: 0.7 per 100,000 VMMCs; 2018: 0.4 per 100,000 VMMC; *p* = 0.02). Most (64%) GIs were partial or complete amputations. All amputations among 10–14 year-olds occurred using the forceps-guided (FG) method, as opposed to the dorsal-slit (DS) method, and three GIs among infants occurred using a Mogen clamp. Of 19 attempted amputation repairs, reattached tissue was viable in four (21%) in the short term. In some cases, inadequate DS method training and being overworked, were found.

**Conclusion:**

Following numerous interventions by PEPFAR and other stakeholders, GIs are decreasing; however, they have not been eliminated and remain a challenge for the VMMC program. Preventing further cases of complete and partial amputation will likely require additional interventions that prevent use of the FG method in young patients and the Mogen clamp in infants. Improving management of GIs is critical to optimizing outcomes.

## Background

Male circumcision (MC) is a preventive procedure that decreases the risk of acquiring HIV in men through heterosexual transmission [1]. It is an essential component of the Joint United Nations Programme on HIV/AIDS strategy for ending AIDS by 2030 in high HIV burden countries in southern and eastern Africa [2]. The U.S. President’s Emergency Plan for AIDS Relief (PEFPAR) has supported ministries of health (MoHs) to provide > 18,900,000 voluntary medical male circumcisions (VMMCs), among males aged 10 years and older,^1^ from 2007 to 2018 in 15 countries in southern and eastern Africa [3], accounting for ~ 80% of all VMMCs for HIV prevention performed worldwide. VMMC services are typically delivered by implementing partners (IPs), which are local or international organizations who receive PEPFAR funding and are overseen by MoHs and U.S. government agencies. All patients provide informed consent for the procedure; consenting for minors adheres to national standards.

Circumcision, when performed by a trained medical provider, is widely considered a safe procedure with low incidence of adverse events (AEs) [4, 5]. Glans injuries (GIs), primarily amputations and lacerations, are rare, severe AEs that can cause permanent disfigurement, functional impairments, and lifelong psychological impact. These AEs can affect VMMC programs through decreased community VMMC acceptance, diminished program reputation, and financial commitments for subsequent care of injured patients.

Three different MC methods – forceps-guided (FG), dorsal slit (DS), and sleeve resection – were used in the seminal randomized-controlled trials that established the preventative effect of MC for HIV. At the outset of the PEPFAR-sponsored VMMC program, most countries adopted the FG method, as it was considered faster and technically easier than other methods. The FG method had been developed and standardized for use in the VMMC program but otherwise is not a widely practiced procedure globally [6]. As the program expanded, the proportion of younger patients (10–14 years old) receiving VMMC steadily increased due to high demand for MC in this age group. However, a World Health Organization (WHO) investigation in 2014 into a series of glans amputations identified use of the FG method in younger patients as the causative factor [7]. With the FG method, the glans is not visualized after the clamp is placed on the foreskin and, because the glans may not be discernably palpable in patients with immature penile anatomy, there is a risk of accidental clamping and injuring it when a scalpel cuts the foreskin. Following its investigation, in July 2014, WHO issued an information notice warning about the risks of using FG method in young patients [7].

Shortly after WHO notified countries about the link between FG method and GIs, PEPFAR instituted policy in 2014 prohibiting FG method use in patients with immature penile anatomy in PEPFAR-supported programs [8, 9]. To promote this shift, PEPFAR supported extensive retraining and mentoring of providers and ensured instruments needed for DS method were widely available. Furthermore, IPs were required to sign written statements agreeing to adhere to PEPFAR policy, which included discrete enforceable actions for violations (e.g., suspension of providers and/or IPs). Ultimately, to achieve adherence and simplify practice, some MoHs with PEPFAR-supported VMMC programs phased out use of FG method among patients of all ages.

Anticipating the limits of policy measures alone, PEPFAR also initiated surveillance through mandatory reporting of GIs. All reported cases were investigated and the findings used to guide corrective action. PEPFAR-supported internal and external quality assurance activities specifically searched for continued FG method use in patients aged < 15 years. PEPFAR conducted educational outreach to stakeholders beyond just PEPFAR grantees, collaborated with WHO on normative guidance to eliminate GIs, and developed clinical guidance on prevention and management of GIs [10]. Lastly, PEPFAR financially supported the medical care of patients experiencing GIs.

Despite these numerous interventions by PEPFAR, a small number of GI cases have continued to occur. Thus, preventing further cases will likely require additional interventions. The purpose of this case series report is to describe cases of GI reported in PEPFAR-supported VMMC programs, assess for factors resulting in better clinical outcomes, identify missing data to improve future investigations, generate hypotheses on root and contributing causes of these AEs, and guide potential interventions to eliminate GIs.

## Methods

### Data source

PEPFAR routinely collects information on several types of severe AEs, including GIs, through the Notifiable Adverse Event Reporting System (NAERS) [11, 12]. NAERS, initiated in 2014 to monitor 30-day post-procedure mortality within PEPFAR-supported VMMC programs, expanded in 2015 to include other severe AEs, including GIs. Under NAERS, these severe AEs must be reported to in-country PEPFAR representatives by IP staff as soon as they learn of the event. An investigation ensues, with the in-country PEPFAR staff reviewing medical records and interviewing IP staff, involved providers, and the VMMC patient. This information is synthesized and submitted on a standardized AE report form, typically within two weeks of AE notification.

From 2015 through 2018, AE investigations submitted to NAERS involved completion of three standard forms in series. The first, completed by the IP contains basic information such as MC and AE date and site. The second, completed by in-country PEPFAR staff, provides clinical information surrounding the circumcision, the AE, and the subsequent management. The third, completed by a PEPFAR consulting physician acting as an objective reviewer, summarizes clinical information, determines relatedness of the AE to the VMMC services provided, and gives recommendations for improving care as applicable.

### Data collection

For all GIs reported to PEPFAR, patient demographics, provider and site characteristics, procedure details, GI management and outcome, and completeness of the NAERS report were reviewed. Although all fields in the investigation form are required, some are straightforward and specific whereas others are longer narrative fields. Variables collected from information appearing in specified fields was available more often than variables collected from information in narrative fields. For example, “surgical method,” a specified field that indicates use of the FG or DS method, was always available, whereas the open-ended field “narrative of clinical course” only sometimes contained information on variables such as provider experience or time to reach a referral facility. While the overall availability and completeness of reporting forms improved with time, only 42% of cases had all three forms available. Data collection and analysis plans were reviewed in accordance with CDC human research protection procedures and was determined to be nonresearch.

### Definitions

Injuries to the glans during circumcision can either be lacerations with no separate piece of tissue, or amputations where there is a completely separated piece containing either the entire glans or a portion of it (i.e., complete or partial amputation). Provider training was classified as inadequate if there was documentation of a lack of, or insufficient, training in the DS method (e.g., receiving didactic instruction on DS method but no practicum). Heavy workload was considered when investigators indicated that high workload may have contributed to GI occurrence. A possible attempt to conceal was noted if the investigator suspected or confirmed inaccurate documentation (e.g., a provider documenting DS when a FG was actually performed) or if the AE was not reported to PEPFAR as required. Handling of severed tissue was dichotomized based on whether or not it was treated as recommended in standard guidelines– wrapped and transported in sterile gauze (preferably saline soaked) [10]. Time to reach referral facility was defined as time from injury to arrival at a health care facility where definitive care could be delivered (note, most GIs require referral although minor GIs—such as a small laceration—that can be rapidly repaired by a competent provider at the VMMC site do not necessitate referral); time from injury to attempted repair was also collected. While repair should be done as quickly as possible by a competent provider, within six hours is generally considered the goal for reperfusion of ischemic tissue. Short-term outcome was determined based on whether or not the injured tissue remained viable, or was healing without noted deficiency, at the latest NAERS entry; while long-term outcome was appearance and function of the penis ≥one year post-injury.

### Data analysis

Descriptive analysis was performed using frequencies for categorical variables and measures of central tendency for continuous variables. The rate of GI was calculated by dividing the total number of injuries by the number of circumcisions performed in patients under age 15 and expressed as GIs per 100,000 circumcisions in this age group. Exact Poisson 95% confidence intervals for GI rates were calculated. The trend in the GI rate across years was evaluated by fitting a Poisson regression model with a robust variance estimator using Stata software version 14.1. Because of missing information, as well as differences in the management of each case, denominators vary between variables; this is noted where it occurred.

## Results

**Fig. 1:**
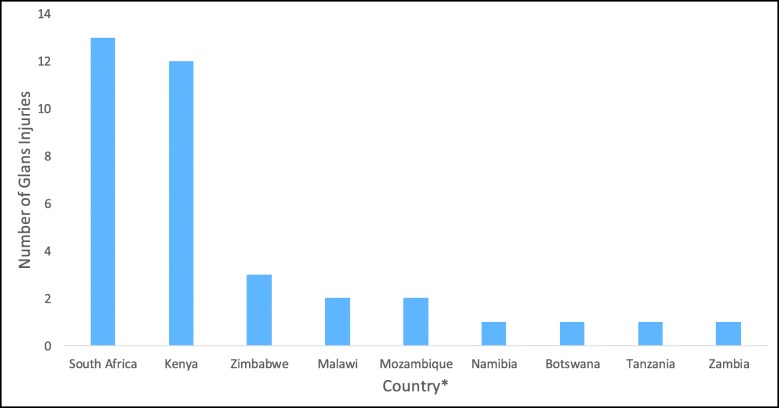
Number of glans injuries reported to PEPFAR by country, 2015–2018, N = 36. During 2015–2018, 12.8 million male circumcisions were performed in PEPFAR-supported VMMC programs, 11.0 million of these had a known age recorded, of which 5.3 million were among patients aged < 15 years. * eSwatini, Ethiopia, Lesotho, Rwanda, and Uganda reported no GIs over this time period

From 2015 through 2018, 36 GIs, all in patients < 15 years old, were reported across PEPFAR-supported VMMC programs in nine countries (three of these injuries occurred during 2014 but were not reported until 2015 and are excluded from the rate calculation). South Africa and Kenya combined to account for 69% of the total number of GIs, while five countries reported none (Fig. 1). The annual rate of GIs per 100,000 VMMCs performed in patients < 15 years old decreased over time for both GIs related to any procedure method (*p* = 0.02) and those related to FG method use (*p* < 0.01) (Table 1). The modeled rate of glans injury related to FG method decreased 26.1% (95% CI: 9.2%–39.9%) per year.

**Table 1:** Rate of glans injuries per 100,000 VMMCs performed in patients less than 15 years old

Year	Any glans injury			Glans injury related to FG		
	Number of injuries	Number of circumcisions age 0–14	Number of injuries per 100,000 circumcisions (95% CI)^b^	Number of injuries	Number of circumcisions age 10–14	Number of injuries per 100,000 circumcisions (95% CI)^c^
2015^a^	8	1,099,859	0.73 (0.31–1.43)	7	1,060,878	0.66 (0.26–1.36)
2016	7	963,067	0.73 (0.29–1.50)	7	953,357	0.73 (0.30–1.51)
2017	11	1,572,079	0.70 (0.35–1.25)	8	1,560,375	0.51 (0.22–1.01)
2018	7	1,689,178	0.41 (0.17–0.85)	4	1,672,993	0.24 (0.07–0.61)
Overall	33	5,324,183	0.62 (0.43–0.87)	26	5,247,603	0.50 (0.32–073)

In July 2014, WHO announced the risk of glans injury with FG method in males 10–14 years. PEPFAR followed shortly thereafter disallowing FG use in this age group. Data on glans injury rate prior to 2015 is not available

3 glans injuries reported retrospectively from 2014 were excluded

The number of PEPFAR notifiable AEs, which include death, glans injuries, tetanus, any AE resulting in permanent deformity or disability, and any AE requiring hospital admission for 3 days or more reported each year increased during this time period. This is suspected to correspond with improved reporting capacity to PEPFAR within countries implementing VMMC. However, although reporting to PEPFAR is mandatory, ascertainment of NAERS is not known

^a^2015 includes an extra quarter of VMMCs in the denominator, corresponding to Oct-Dec 2014 (i.e., FY2015Q1). This data was not disaggregated at the time. Thus, the actual rate for 2015 is slightly higher than shown, although unknown to what degree

^b^*P*-value = 0.02 for testing a trend in rates across years

^c^*P*-value < 0.01 for testing a trend in rates across years

*FG* forceps-guided method; *CI* confidence interval

Patient age ranged from 0 to 14 years and patients aged 10 years were most commonly injured (19/36, 53%) (Table 2). The FG method was used in 29/36 (81%), 4/36 (11%) reported use of the DS method, and 3/36 (8%) occurred with use of the Mogen clamp device (all during early infant [< 60 days old] circumcisions) (Table 3). Partial or complete glans amputations, reported in 23/36 (64%), were the most common types of GI. DS use did not lead to amputation in any case. Urethral injury was documented in 22/36 (61%), with 4/36 (11%) uninjured and the remainder unknown (28%). When time of day the GI occurred was documented, 21/28 (75%) happened in the afternoon (Table 2).

**Table 2:** Circumstances surrounding glans injuries occurring within PEPFAR-supported VMMC programs, 2015–2018

	Number (%), ***N*** = 36
**Patient age in years**	
Median (25, 75%)	10 (10, 11)
Mean (range)	10 (< 1–14)
Mode (*n* = 19)	10
**Time of day glans injury occurred**	
8 am – Noon	7 (19)
Noon – 4 pm	13 (36)
After 4 pm	8 (22)
Unknown	8 (12)
**Cadre type of VMMC provider**	
Physician	8 (22)
Clinical Officer/ Clinical Associate	8 (22)
Nurse	4 (11)
Other	1 (3)
Unknown	15 (42)
**Training of VMMC provider in DS method**^1^	
Adequate	2 (6)
Inadequate	5 (14)
Unknown	29 (81)
**Reported over-worked environment during glans injury**^2^	
Yes	9 (25)
No mention	21 (58)
Record unavailable	6 (17)
**Possible attempt to conceal information**^3^	
Yes, inaccurate documentation	11 (31)
Yes, failure to report as required	3 (8)
No	16 (44)
Unknown	6 (17)
**Specialty of referral provider**	
Urologist	17 (47)
Pediatric Surgeon	8 (22)
Other Surgeon	2 (6)
Unknown	3 (8)
Not or unknown if referred	6 (17)

^1^Adequate = AE report indicates adequate training for dorsal slit method, inadequate = report indicates that there was a lack of adequate training, unknown = no mention of training in the report

^2^Documentation of a concern that a heavy workload may have been a contributing factor to the glans injury

^3^Documentation of a concern that VMMC provider or clinic attempted to hide or alter evidence concerning the glans injury (for example, documenting DS method when FG method was actually performed)

**Table 3:** Glans injury type by male circumcision method in PEPFAR supported VMMC programs, 2015–2018

MC method	Amputation			Laceration	Strangulation	Unknown type of injury	Total
	Complete	Partial	Degree not specified				
Forceps-guided	9 (31%)	11 (38%)	1 (3%)	3 (10%)	0 (0%)	5 (17%)	29 (100%)
Dorsal slit	0 (0%)	0 (0%)	0 (0%)	3 (75%)	1 (25%)	0 (0%)	4 (100%)
Mogen clamp^a^	0 (0%)	2 (67%)	0 (0%)	0 (0%)	0 (0%)	1 (33%)	3 (100%)
All methods	9 (25%)	13 (36%)	1 (3%)	6 (17%)	1 (3%)	6 (17%)	36 (100%)

^a^Mogen clamp used only in early infant (< 60 days old) circumcisions

Physicians, clinical officers/associates, and nurses were all identified as the primary provider responsible for a GI; however, cadre was unknown in 15 cases. When documented, physicians and clinical officers were most commonly involved (eight cases each). In five of the seven (71%) cases where DS training was mentioned, it was reported inadequate. In 9/36 (25%), heavy workload may have been a contributing factor. For example, one record documented that a single provider completed over 140 circumcisions in a day, not finishing until after midnight. In 14/36 (39%), the report contained evidence that a VMMC provider or site staff may have attempted to hide or alter evidence concerning the glans injury.

**Fig. 2:**
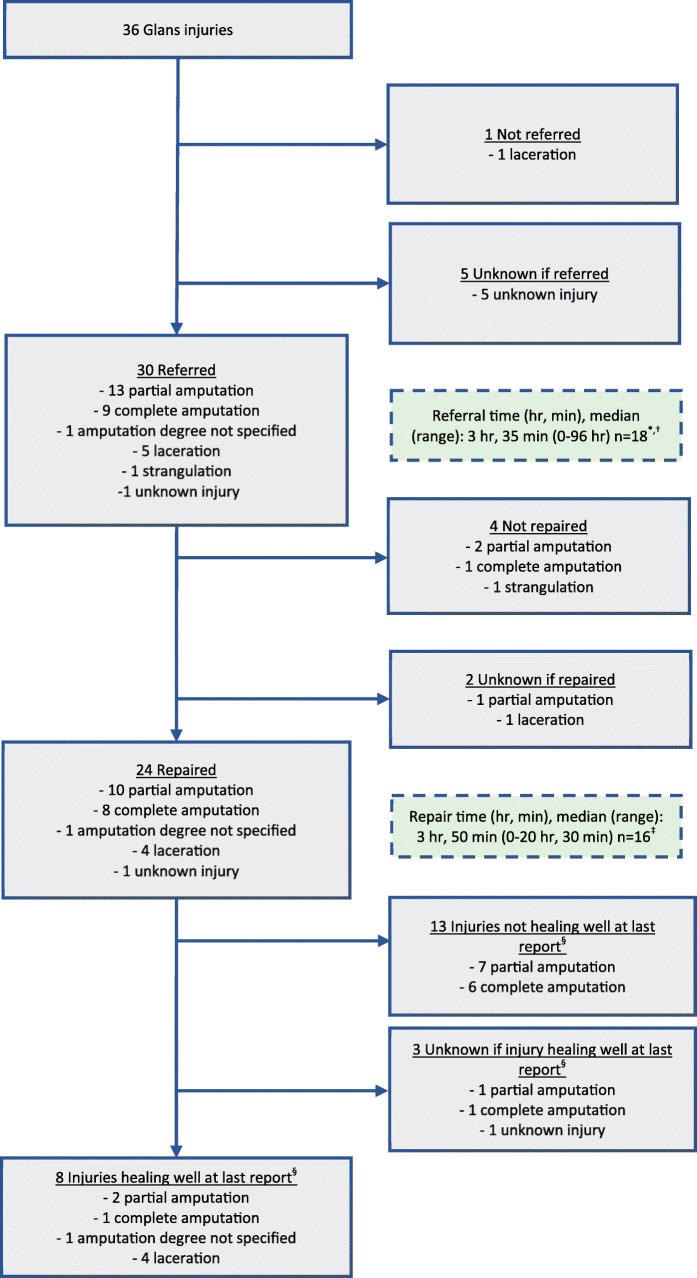
Glans injuries clinical management, PEPFAR-supported VMMC programs, 2015–2018. ^*^Two outliers excluded: one was an amputation not diagnosed for 2 weeks and one was a strangulation injury not diagnosed for 4 weeks. ^†^Ten cases were known to have been referred but referral time was missing. ^‡^Eight cases were known to have been repaired but repair time was missing. ^§^Healing well implies reattached tissue was viable for repaired amputations or healing without documented impairment for repaired lacerations. Since long-term follow-up not available in reports, permanent sequelae such as tissue atrophy, scarring, urethral strictures, urethral fistulae, and altered sensation are unknown.

Once a GI occurred, most patients were referred to a higher level of care for management (30/36, 83%), with urologists being the most common type of accepting physician (17/36, 47%). In only 5/23 (22%) amputations was the severed tissue handled properly (with 5/23 [22%] unknown). The median time to arrival at referral site was 3:35 (range 0–96 h) and the median time to repair was 3:50 (range 0–21 h) (Fig. 2). Of 19 patients with complete or partial amputations who had a known repair attempt, 9 (47%) had it performed within six hours from the time of injury. Four of 30 (13%) patients known to be transferred went to two or more facilities before receiving definitive initial treatment of injury (one patient with unknown number of referral sites). Patients transferred between two or more referral facilities had longer median times to repair (3:35 versus 8:20). Of glans amputation cases known to have undergone repair, only 4/19 (21%) reports documented viability of reattached tissue. No reports contained information on long-term outcome.

## Discussion

During four years of surveillance, PEPFAR detected 36 GIs, or approximately 0·7 GIs per 100,000 VMMCs in patients < 15 years old. Following numerous interventions by PEPFAR, MoHs, and other stakeholders, GIs have decreased, particularly those due to use of the FG method. However, they have not been eliminated and remain a challenge for the VMMC program.

Complete and partial glans amputations are preventable through proper use of a circumcision method that directly visualizes the glans during removal of the foreskin. This case series report found that GIs occurred exclusively among patients < 15 years old, the majority of whom had undergone a FG circumcision, and that the types of GI occurring using the DS technique were lacerations, which are easier to repair and have better outcomes than amputations. Although eliminating use of the FG method and Mogen clamp in patients under age 15 should eliminate complete and partial amputations, use of the DS method will still incur the risk of a glans laceration and other interventions may be needed to minimize this risk. The link between GI and younger age is presumably a reflection of an increased likelihood of injuring a smaller, sexually immature penis during a FG circumcision. This conclusion is further supported by the finding that over half occurred in 10-year-old patients, where the greatest proportion of patients will have immature anatomy. The three GIs in infants occurred with use of the Mogen clamp, which has been the primary method used for the small number of early infant male circumcisions (EIMCs) performed under PEPFAR-supported VMMC programs and is similar to the FG method in that it does not allow visualization of the glans during foreskin removal. Given the experience of FG use in adolescents, PEPFAR has cautioned about use of the Mogen clamp in EIMC [13].

This analysis adds to our current understanding of GIs by providing insight on factors potentially contributing to continued use of FG method in patients aged < 15 years despite multiple interventions to prevent its use. First, it does not appear related to the level of professional training of the circumcising provider. Physicians and clinical officers/associates, who have more overall medical training than nurses, were the most common cadre responsible for GIs when cadre type was known. This is notable considering that VMMC is primarily a nurse-driven program, with a smaller portion of VMMCs performed by other cadres. The discrepancy may result from less procedure-specific competence by these cadres, a relative lower frequency of performing VMMCs because of other clinical duties, and/or reluctance by team members to challenge physicians using unsafe practices. Regarding competence, in the few instances in which provider perception of training adequacy was documented, a majority of providers reported not being adequately trained in the DS procedure.

Being overworked may be a contributing factor by prompting providers to revert to the FG method, which they may have more comfort performing and perceive to be faster. Two findings from this analysis support this hypothesis: multiple reports contained verbiage (such as high case volumes or rushing to complete) suggesting that being overworked was a possible contributing factor, and most GIs occur in the afternoon, an indication that clinics may be running behind schedule (sometimes older patients are intentionally seen first and younger boys have to wait, which could contribute to this finding). This concern about being overworked, along with use of the FG method, contributing to GIs during VMMC was noted in a small case series from South Africa [14]. A safe maximal number of cases per staffing is likely dependent on additional factors such as staffing type and experience, resources, and infrastructure. Programs are encouraged to determine a safe number of procedures that can be done dependent on a site’s composition and resources. One example from South Africa details a team of 3 doctors and 12 nurses being able to circumcise up to 50 patients a day [15]. It is important to document workload in future AE investigations as this may provide quantitative data to inform the investigation and decision making.

Although the priority is prevention of GIs, this analysis showed improvements to post-injury management are needed as well. In the majority of cases where severed tissue handling was documented, providers did not wrap the tissue in saline-soaked sterile gauze or transport in a cool environment as is recommended [10, 16, 17]. Placing the wrapped tissue on, but not in direct contact with, ice should be done when possible as long as this does not delay patient transfer. Additionally, reports rarely mentioned how the provider addressed the cut surface of the proximal stump. Digital pressure to control hemorrhage, rather than cautery or hemostatic sutures, is important for the initial survival of reattached tissue. Performing cautery or suturing not only wastes valuable time, but can impair the provision of nutrients to the reattached tissue which relies on preservation of blood flow to this proximal cut edge. Although, on average, patients were transported to referral centers quickly, many did not undergo repair within six hours. In some cases, delays in repair accompanied multiple transfers between referral centers. The need to minimize ischemic time of severed tissue, and have a reattachment performed as quickly as possible, cannot be overemphasized. Having valid and up-to-date referral processes in place before a GI occurs is key to decreasing times to referral and repair. Immediately available flowcharts describing proper tissue handling, providing referring physician contact information, and emphasizing the importance of timely repair could prompt appropriate decisions during a stressful and chaotic time.

Single cases [18–20], or small series [16, 17], have documented successful reattachments following glans amputations, typically after EIMC. Given the rarity of GIs, a likely reporting bias favoring successful reattachments, and a lack of generalizability of early infant repairs to older children, an expected reattachment success rate is unknown. In this case series, less than a quarter of reattachment attempts were successful in the short-term. Although, short-term success does not preclude later-onset complications such as urethral strictures or fistulas, altered glans sensitivity, and glans scarring or atrophy. Optimized management during the immediate post-injury period gives the best chance at long-term success, but is still unlikely to result in completely normal appearance and function. This dismal rate of reattachment success speaks to the importance of prevention.

A possible attempt to conceal either the GI itself, or certain details about the case, is a particularly worrisome finding. It suggests awareness of, and disregard for, policy intended to improve safety. These cases led to immediate termination of the provider once identified. Transparency in all facets of an AE investigation is vital to allow analysis of root and contributing causes. The temptation to oversimplify the cause of an AE, to narrow it down to one component of care, must be avoided. Although use of the FG method may be the immediate cause of GIs, this analysis suggests there may be multiple factors influencing a provider’s decision to use the FG method such as: lack of comfort performing the DS method, overwork, and increased demand from an at-risk subpopulation of young patients. If providers use the FG method against policy, there are likely one or more reasons driving this decision that they deem more important than policy adherence.

This analysis has several limitations. Although demographic information was available for most cases, many were missing important information from the narrative summaries. Additionally, some variables (e.g., attempt to conceal) involve judgement by the investigator of a provider’s intent. Some key areas where greater detail is important include: adequacy of DS training, referral and repair times, tissue handling, and outcomes. Furthermore, results may be affected by reporting bias to the NAERS passive reporting system. Because the association between FG method in young patients and GI was known during the period examined, providers misusing this method might have been reluctant to disclose its use. Next, while underreporting of AEs is suspected, it is unknown if systematic differences occur within or across countries or regions that inhibit comparability and generalizability of findings. Small sample size and missing data limited our ability to statistically associate practice variations with outcome. Although the majority of GIs occurred to 10 year-old patients, age breakdown by year was not available for the 10–14 year-old group to allow comparison with overall VMMCs per age. Finally, conclusions from this case series should be viewed as hypothesis-generating and would require comparison to controls to determine significance. Still, even without controls, findings like all cases being under age 15 and all amputations among 10–14 year-olds occurring with FG stand out, especially when combined with biologic plausibility, and support interventions taken by PEPFAR and other stakeholders.

Upstream interventions are more effective to produce desired outcomes than those relying on a provider’s individual decision [21]. For example, having a national policy mandating use of the DS method in patients under age 15, but allowing the FG method in adult patients, is an administrative change that still relies on a provider’s adherence to that policy. Because the FG and DS methods each require the use of a small number of unique, key instruments, it is possible to remove those necessary for performing FG procedures from a site. Exclusively procuring VMMC procedure kits that do not contain the few instruments needed to perform a FG, but only those necessary for a DS circumcision, would make the FG method use highly unlikely. Of course, such a change would have to follow adoption of a MoH policy of DS method for all ages. In Uganda, which hosts the largest VMMC program supported by PEPFAR, the FG method has never been practiced and no GI has ever been reported. However, Zambia, also a DS-only program since inception, reported a GI in 2018 when a provider anomalously used the FG method in a 10-year-old patient. This serves as an example that prevention of GIs will likely require multiple interventions at different levels, potentially targeting policy, equipment, training, site administration (e.g., ensuring an appropriate caseload for staffing levels), and individual provider behavior.

Finally, this analysis identified the need for better data collection. Conducting a full root cause analysis can gather candid feedback from site staff to identify immediate and contributing causes. A standardized supplemental GI investigation form, in addition to the standard NAERS report, can capture critical information needed to understand the circumstances around GI occurrence, identify management deficiencies, and guide performance improvement interventions.

## Conclusions

PEPFAR has supported MoHs to take multiple steps to prevent GIs, followed by evidence of improvements in safety. However, additional measures are needed to achieve the goal of zero GIs. Continued use of the FG method is the likely immediate cause of the majority of these injuries although it is important to note glans lacerations can still occur even with use of the DS method. Recognizing the complexity of contributing factors is essential and this analysis suggests that a multi-faceted approach, aimed at both the program and provider levels, may be best suited to eliminate GIs in this vulnerable population.

## Data Availability

The dataset used during the present study is available from the corresponding author on reasonable request.

## Abbreviations

MC: Male circumcision
PEPFAR: President’s Emergency Plan for AIDS Relief
MoH: Ministry of Health
VMMC: Voluntary medical male circumcision
IP: Implementing partner
AE: Adverse event
GI: Glans injury
FG: Forceps-guided
DS: Dorsal slit
WHO: World Health Organization
NAERS: Notifiable Adverse Event Reporting System

## Footnotes

1 PEPFAR has also supported a small number of early infant (0–60 day) male circumcisions in select countries
